# Supplementary material for: Preparation of Biochar with Developed Mesoporous Structure from Poplar Leaf Activated by KHCO3 and Its Efficient Adsorption of Oxytetracycline Hydrochloride
Source: Molecules. 2023 Apr 3;28(7):3188. doi: 10.3390/molecules28073188 (PMC10096365; doi:10.3390/molecules28073188)
Supplement: Supplementary file 1 [file molecules-28-03188-s001.zip › molecules-2316494-supplementary.pdf]

Article

Preparation of biochar with Developed Mesoporous Structure from Poplar Leaf Activated by KHCO3 and Its Efficient Adsorption of Oxytetracycline Hydrochloride

Zhenhua Wei, Chao Hou, Zhishuo Gao, Luolin Wang, Chuansheng Yang, Yudong Li, Kun Liu * and Yongbin Sun *

Institute of Optical Functional Materials for Biomedical Imaging, School of Chemistry and Pharmaceutical Engineering, Shandong First Medical University & Shandong Academy of Medical Sciences, Taian 271016, China; zhwei@sdfmu.edu.cn (Z.W.); hczhentao1@163.com (C.H.)

* Correspondence: liukun2436@126.com (K.L.), sunyongbin6033@163.com (Y.S.)

Calculation method for specific surface area and pore diameter

(1) The total specific surface area is calculated using the BET method, and the equation is as follows.

$$\frac{p}{V(p_0-p)}=\frac{1}{V_m C}+\frac{C-1}{V_m C}\times\frac{p}{p_0} \tag{S1}$$

Where p: equilibrium pressure of adsorbed gas at adsorption temperature; p_0: saturated vapor pressure; V: total volume of adsorbed gas at equilibrium pressure p; V_m: volume of adsorbed gas required when the sample surface is exactly completely covered; C: a constant related to adsorption.

In the above equation, $\frac{p}{V(p_0-p)}$ is the ordinate, $\frac{p}{p_0}$ is the abscissa, the slope is A (=$\frac{C-1}{V_m C}$), and the intercept is B (=$\frac{1}{V_m C}$).

$$V_m=\frac{1}{A+B}$$

Assume that the adsorbed gas is nitrogen, the total specific surface area is calculated using the following equation:

$$S=\frac{4.35\times V_m}{m} \tag{S2}$$

Where m: the weight of sample.

(2) The mesoporous specific surface area is calculated using the BJH method [56].

Assume that the adsorbed gas is nitrogen, and all holes are cylindrical, the radius is calculated using the following equation:

$$r=-\frac{0.953}{\ln\frac{p}{p_0}}+\frac{0.354}{\left(\frac{-5}{\ln\frac{p}{p_0}}\right)^{\frac{1}{3}}} \tag{S3}$$

The diameter $\boldsymbol{d}$ = $\mathbf{2}\boldsymbol{r}$.

Assume that all holes are cylindrical, the specific surface area is calculated using the following equation:

$$S=\frac{8\times V}{a+b} \tag{S4}$$

Where a–b: the measured diameter range, V: nitrogen adsorption volume within this diameter range.

The mesoporous specific surface area is calculated by accumulating the specific surface area in the range of 2–50 nm.

Supporting figures and tables

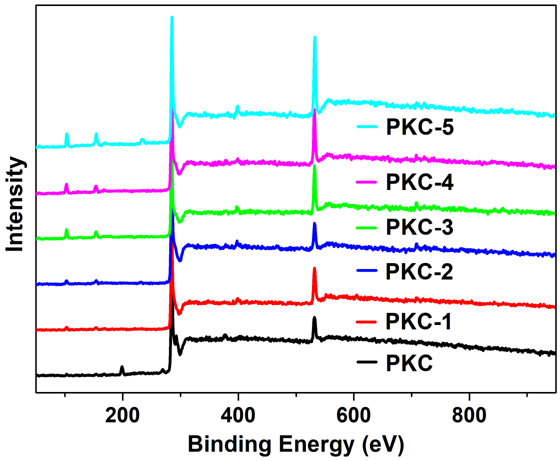

Figure S1. XPS full spectra of biochars prepared from poplar leaves with $KHCO_3$ as activator.

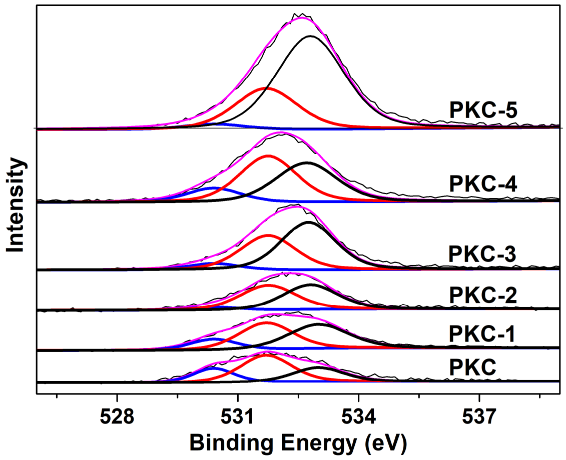

Figure S2. High-resolution O1s XPS spectra of biochars.

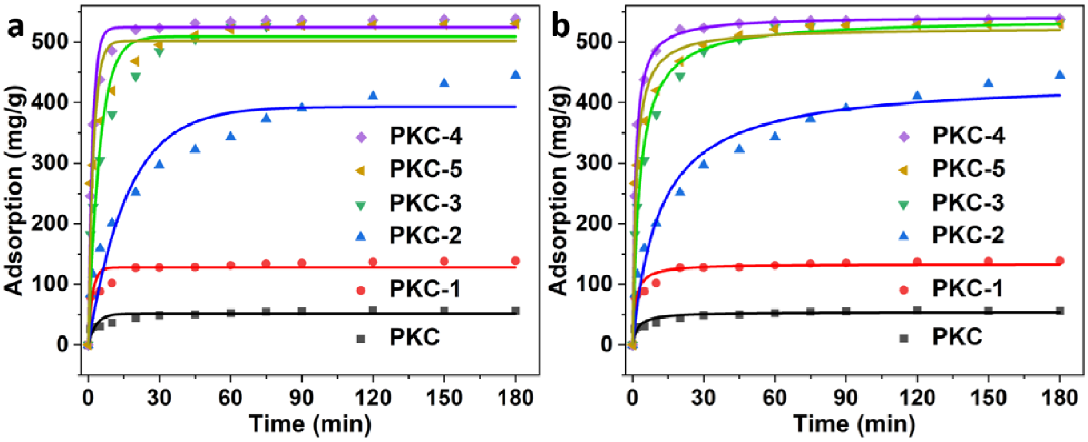

Figure S3. (**a**) Pseudo-first-order kinetic model (solid line) and (**b**) pseudo-second-order kinetic model (solid line) for the adsorption capacities of biochars at different times.

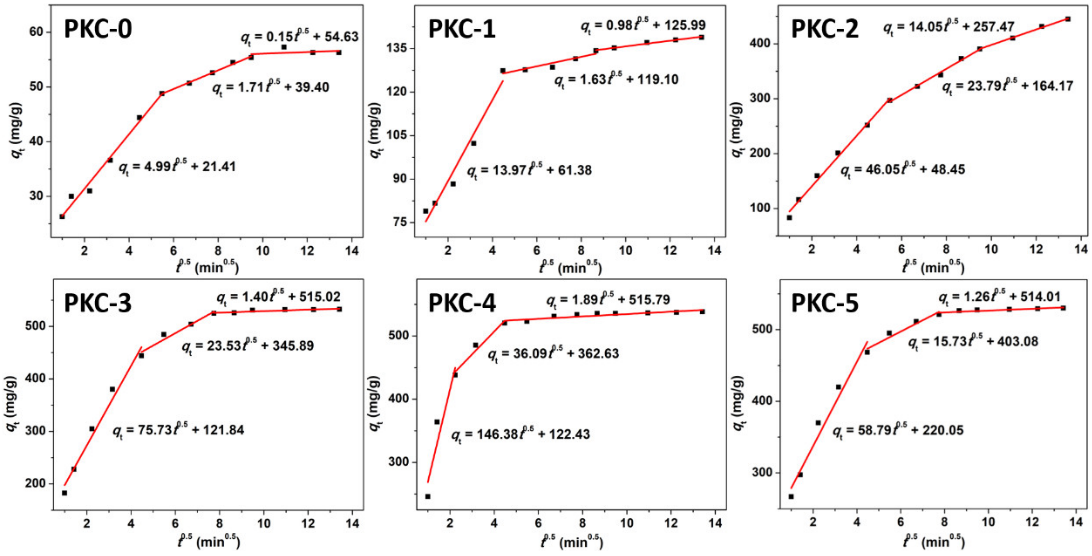

Figure S4. Linear intra-particle diffusion fittings for OTC on biochars.

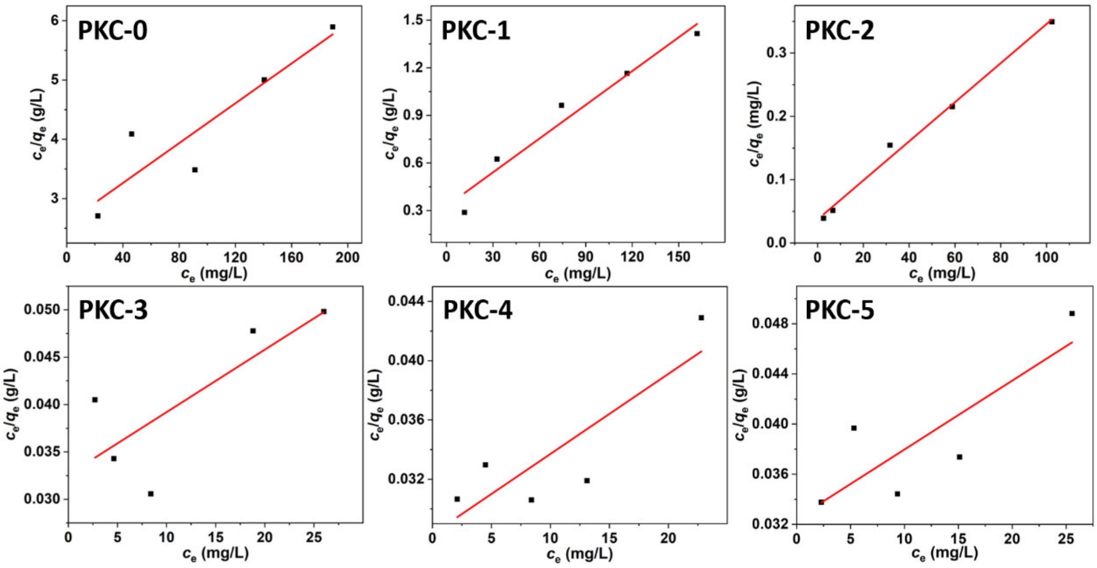

Figure S5. Langmuir model (solid line) for the equilibrium adsorption capacities of biochars at different initial OTC concentration.

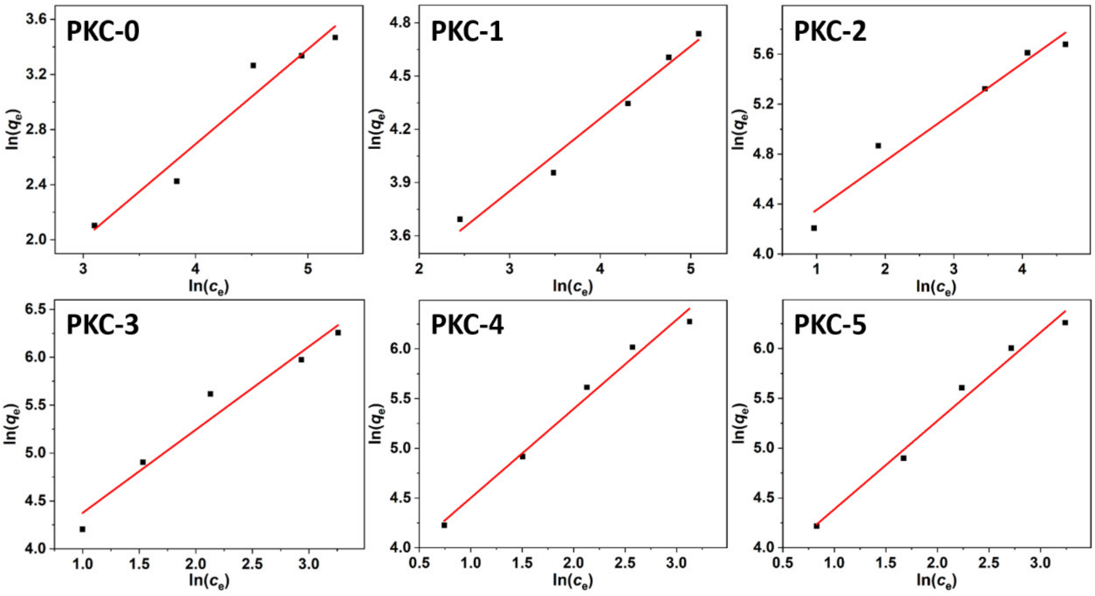

Figure S6. Freundlich model (solid line) for the equilibrium adsorption capacities of biochars at different initial OTC concentration.

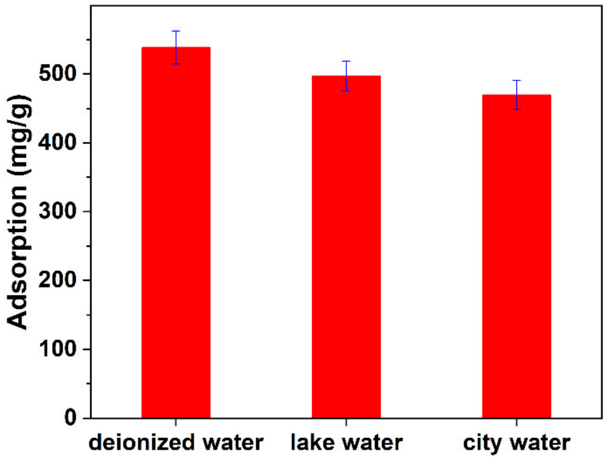

Figure S7. Adsorption capacity of PKC-4 on OTC in different water matrices.

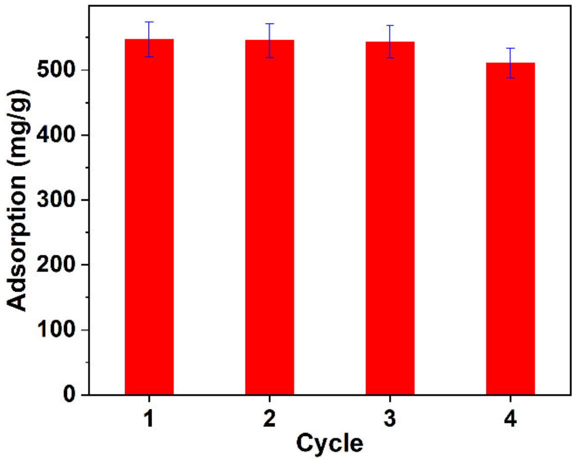

Figure S8. Cyclic adsorption performance of PKC-4 for OTC.

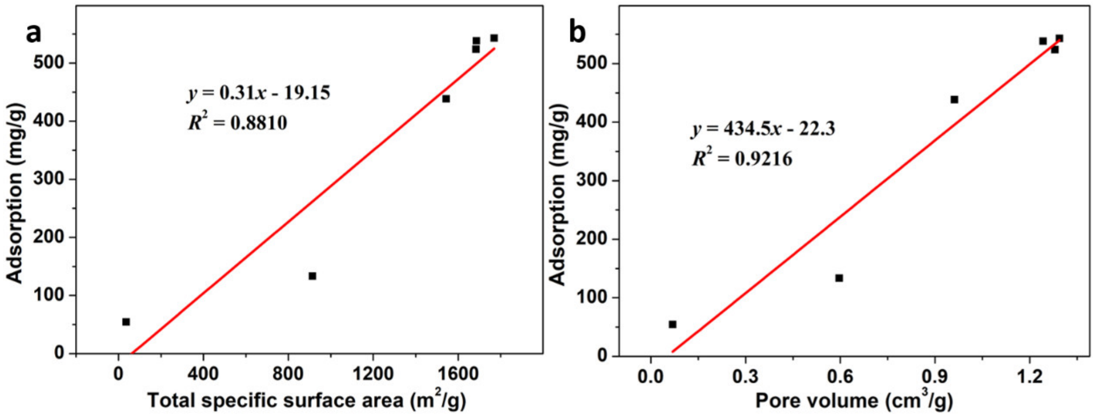

Figure S9. Correlation between the total specific surface area, pore volume and the adsorption capacity of PKC-4.

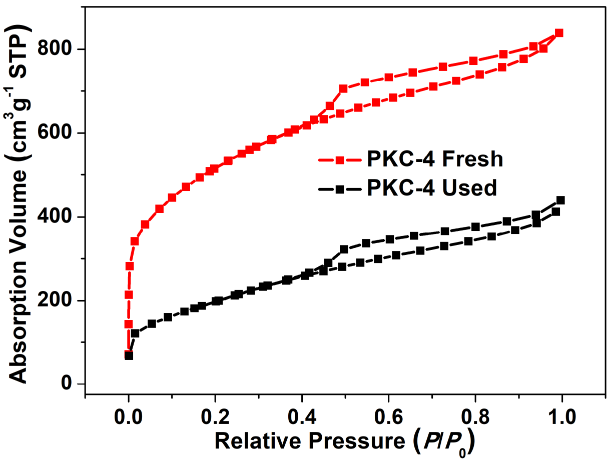

Figure S10. Nitrogen adsorption-desorption isotherms of fresh PKC-4 and used PKC-4.

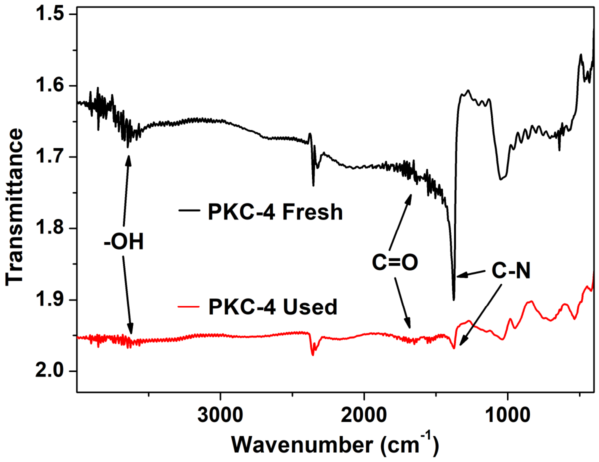

Figure S11. FTIR spectra of fresh PKC-4 and used PKC-4.

Table S1. Yields of the biochars prepared from poplar leaves with $KHCO_3$ as activator.

Biochar	PKC-0	PKC-1	PKC-2	PKC-3	PKC-4	PKC-5
Yield (wt%)	21.3	17.5	14.3	11.7	10.2	9.1

Table S2. The I_G/I_D ratios of biochars.

Biochar	PKC-0	PKC-1	PKC-2	PKC-3	PKC-4	PKC-5
I_G/I_D ratios	0.993	0.977	0.949	0.896	0.894	0.850

Table S3. The elemental atomic percentage of biochars analyzed by XPS.

Biochar	Elemental atomic percentage (%)		
	C	N	O
PKC-0	88.62	2.93	8.45
PKC-1	86.3	2.05	11.66
PKC-2	84.33	2.07	13.6
PKC-3	83.36	2.06	14.58
PKC-4	82	2.87	15.13
PKC-5	75.21	3.73	21.07

Table S4. A comparison between the adsorption performance over PKC-4 and other samples for the adsorption of OTC.

Sample	Adsorption (mg/g)	Reference
SFB2-900	730	[57]
PKC-4	543	This work
FeNiZn-LBC	476	[44]
ZnFe-LDH/MBC	427	[58]
ZIF-8	312	[59]
nZVI-HBC	197	[60]
CuFe2O4/NiMgAl-LDH	192	[61]
Fe3O4@C@TiO2	87	[62]
Zeolite/Fe3O4	83	[63]

Table S5. Specific surface areas and porosity characteristics of fresh PKC-4 and used PKC-4.

Biochar	Total specific surface area (m^2/g)	Mesoporous specific surface area (m^2/g)	S_{meso}/S_{total}	Pore volume (cm^3/g)	Average pore size (nm)
PKC-4 fresh	1769.7	1140.0	64.4%	1.2945	2.95
PKC-4 used	725.5	702.4	96.8%	0.6549	3.61

References are listed in the main file in the back matter.
